# Engineering 3D approaches to model the dynamic microenvironments of cancer bone metastasis

**DOI:** 10.1038/s41413-018-0008-9

**Published:** 2018-02-26

**Authors:** Han Qiao, Tingting Tang

**Affiliations:** Shanghai Key Laboratory of Orthopaedic Implants, Department of Orthopaedic Surgery, Shanghai Ninth People’s Hospital, Shanghai Jiao Tong University School of Medicine, Shanghai, 200011 China

## Abstract

Cancer metastasis to bone is a three-dimensional (3D), multistep, dynamic process that requires the sequential involvement of three microenvironments, namely, the primary tumour microenvironment, the circulation microenvironment and the bone microenvironment. Engineered 3D approaches allow for a vivid recapitulation of in vivo cancerous microenvironments in vitro, in which the biological behaviours of cancer cells can be assessed under different metastatic conditions. Therefore, modelling bone metastasis microenvironments with 3D cultures is imperative for advancing cancer research and anti-cancer treatment strategies. In this review, multicellular tumour spheroids and bioreactors, tissue engineering constructs and scaffolds, microfluidic systems and 3D bioprinting technology are discussed to explore the progression of the 3D engineering approaches used to model the three microenvironments of bone metastasis. We aim to provide new insights into cancer biology and advance the translation of new therapies for bone metastasis.

## Oncology: Modelling bone metastasis in 3-dimensions

Better 3D models are needed to understand the entire process of bone cancer metastasis, if new treatments are to be developed. Bone metastasis is a major complication of several common cancers, yet its biology is poorly understood - in part, because conventional cell culture systems have failed to replicate the 3D microenvironment of the body, where cancer cells dynamically interact with healthy cells and the extracellular matrix. To address this, 3D models of the primary tumour microenvironment, circulation microenvironment and bone microenvironment are needed, suggest Han Qiao and Tingting Tang at Shanghai Jiao Tong University School of Medicine in China. They describe promising 3D approaches, including multicellular tumour spheroids, tissue engineering constructs, microfluidic systems, and 3D bioprinting, but stress that none of these currently recapitulates the entire process of metastasis in a single culture system.

## Introduction

Bone metastasis is the major complication of advanced osteotropic cancers, including breast cancer (BC), prostate cancer (PC), lung cancer (LC) and multiple myeloma (MM). These metastases can cause significant morbidity due to skeletal-related events including pathological fracture, spinal cord compression, bone pain and hypercalcemia. In addition, metastatic bone lesions contribute to a poor prognosis, despite current therapeutic strategies.^1–3^ Hence, it is imperative to develop novel effective treatments for bone metastasis through a better understanding of malignant bone metastases in the clinical setting. Cancer cells naturally inhabit a three-dimensional (3D) architecture within host microenvironments. Currently, two-dimensional (2D) culture biosystems fail to consider the dynamic interactions between cancer cells and the microenvironment, and these systems differ from actual 3D biostates in regulating the genotypic and phenotypic bioactivity of malignant cells. Studies involving 3D biosystems over the past several decades have significantly bridged the gap between 2D culturing patterns and in vivo animal models.^4–6^ Hence, it is important to take advantage of spatial approaches in bone metastasis research to emphasise the dynamic dialogue between cell–cell and cell–extracellular matrix (ECM) interactions.

**Fig. 1:**
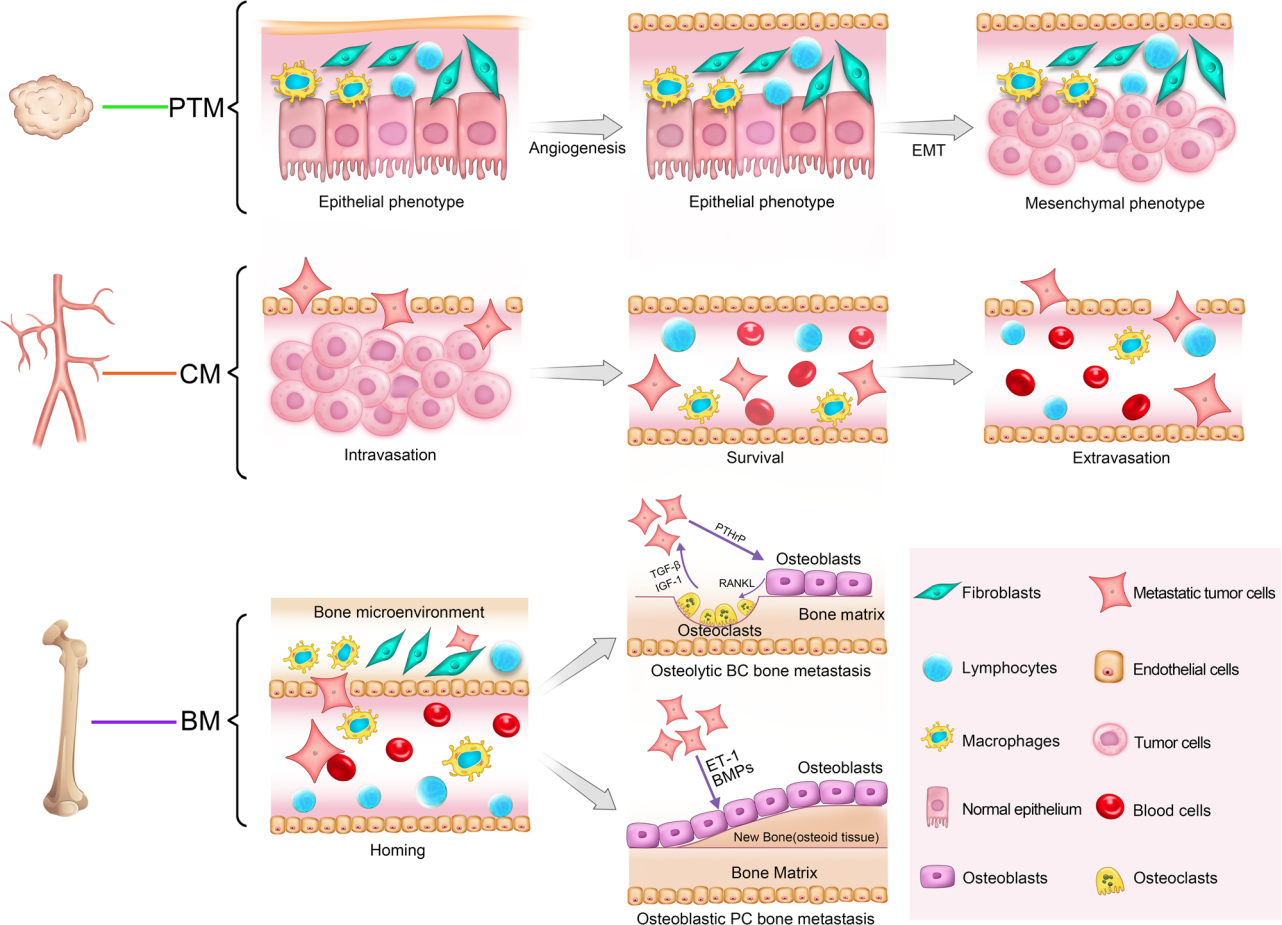
Illustrations of three metastatic microenvironments during osteolytic and osteoblastic cancer bone metastases

To date, the evolution of malignant bone metastasis has classically been characterised as a dynamic multistep process, namely, the invasion-metastasis cascade, in which cancer cells undergo a sequential journey of primary tumour transformation, local invasion, intravasation, survival in circulation, extravasation and metastatic colonisation in a distant bone microenvironment.^7^ Stephen Paget proposed the “seed and soil” hypothesis,^8^ which suggests that cancer cell metastasis is akin to the dissemination of plant seeds. To better understand the underlying biology of bone metastasis, separation of the complex cascade into several more explicit and foreseeable systems is required. Herein, we expand the connotation of “soil” to a wider range consisting of the following three microenvironments during cancer bone metastasis: the primary tumour microenvironment (PTM), circulation microenvironment (CM) and bone microenvironment (BM) (Fig. 1). Establishing the most representative 3D microenvironment is imperative and requires a comprehensive understanding of the application of 3D approaches in cancer research.

## Overview of bone metastatic microenvironments

In the PTM, the advent of a compatible metastasis frequently occurs in osteotropic tumour cells with limitless proliferative capability in primary sites; in this process, angiogenesis is critically important.^9^ When there are metabolic stresses on the tumour cells, the equilibrium between pro- and anti-angiogenic factors is altered, leading to recruitment of endothelial cells and fibroblasts, which form new vessels from the surrounding stroma.^10^ Angiogenesis not only satisfies the heightened metabolic needs of cancer cells but also supports avenues for local infiltration and foreign dissemination. Furthermore, another critical event in promoting cancer cell metastasis is epithelial–mesenchymal transition (EMT). In response to various extracellular EMT-inducing signals, potential metastatic cells orchestrate invasion-promoting molecular, cellular and morphological changes through a cellular transformation from an epithelial phenotype with apical-basal polarisation to a mesenchymal phenotype with high motility capability and a spindle shape.^11^ Then, the cells access vascular systems with the assistance of newly formed microcapillaries, resulting in the onset of subsequent cellular events in the CM.

The activity of cancer cells in the CM begins with intravasation and ends with extravasation. Intravasation involves a series of dynamic interactions between malignant cells and the microenvironment, such as decreased intercellular adhesion, increased cytoskeletal motility, active ECM remodelling and widened endothelial gaps, which accelerate the migratory pace of cancer cells to distant bone. Tumour cells that breach the normal vascular endothelium become circulating tumour cells (CTCs). With the substantial cell attrition induced by trans-membrane migration, fewer than 1% of original CTCs are capable of successful colonization in secondary sites.^12^ A number of immune cells, particularly macrophages and T-lymphocytes, obliterate CTCs within the CM.^13^ One example of how CTCs escape cell death in the CM is by inhibiting anoikis. Normally, the loss of cell–cell and cell–ECM interactions in migratory cells leads to cell apoptosis, but CTCs can overexpress a series of surface receptors to activate pro-survival pathways,^14^ resulting in their ability to survive in the CM through anoikis resistance.

The BM of bone tissues provides fertile “soil” for circulating osteotropic cancer cells to settle and populate. Metastatic bone lesions can generally be classified into osteolytic (such as in BC bone metastases) and osteoblastic (such as in PC bone metastases) microenvironments, which have distinct radiographic appearances. Osteoclasts are multinucleated cells derived from precursor osteoclasts of the monocyte-macrophage lineage. These cells dissolve calcium phosphate crystals and degrade ECM to demineralise bone structure by releasing relevant functional proteases. Osteoblasts are specialised, well-differentiated mononucleated cells derived from mesenchymal stem cells (MSCs) in the bone marrow cavity. These cells produce calcium phosphate crystals and generate newly formed ECM into matrix interstices for bone remineralization.^15^ Therefore, bone homoeostasis is maintained by the equilibrium between osteoblasts and osteoclasts, while its disruption may convert the BM from normal physiological niches to pathological metastatic niches. CTCs that enter the BM become disseminated tumour cells (DTCs), which can persist in dormancy for several years.^16^ Under conditions of increased stress/load, decreased immunity and stimulation by molecular signals, dormant DTCs progress to macrometastases, confirming the clinical significance of eliminating dormant cells in the BM to achieve long-term remission and overcome oncotherapy resistance.

Although osteocytes are the most abundant bone cells in bone tissues compared with osteoclasts and osteoblasts, little is known about their roles in osteotropic cancer bone metastasis.^17^ Osteocytes participate in osteoclastogenesis and osteoblastogenesis by secreting receptor activator of nuclear kappa B ligand (RANKL), osteoprotegerin (OPG), macrophage colony stimulating factor (M-CSF) and sclerostin.^18^ Therefore, targeting osteocytes to inhibit osteolytic RANKL expression abrogates early BC bone metastasis.^19^ In addition, serving as mechanical sensor cells, osteocytes react to shear stress and pressure from bone metastases, contributing to reciprocal tumour growth and osteoclast formation.^20,21^

For BC bone metastasis, once circulating cancer cells manage to survive in distant osseous tissues, cellular and extracellular components adapt to create a compatible niche by initiating a reciprocal vicious cycle between malignant cells and the bone microenvironment. This cycle in the BM involves mutual crosstalk between cancer cells and the microenvironment, and represents the core implication of 3D approaches in cancer bone metastasis research. With respect to PC bone metastasis, tumour cells generate several growth factors, such as endothelin 1 (ET-1) and bone morphogenetic proteins (BMPs), that stimulate osteoblastic progenitor cell recruitment and maturation to produce new pathological bone,^22^ namely, immature mineralised bone (osteoid) around metastasis sites.

Collectively, numerous key players involved in bone metastasis, including biotic cellular components, such as tumour cells, endothelial cells, immune cells, osteoblasts, osteoclasts and osteocytes, and abiotic factors, such as growth factors and mechanical support, have been implicated in making metastatic microenvironments permissive for malignant cells.^23^ Cancer cells within metastatic bone microenvironments undergo a series of genetic and morphologic transformations that are stimulated by the extracellular microenvironment.^24^ However, with respect to 3D approaches, modelling the entire metastasis process within individual biosystems is difficult because metastatic microenvironments have varying yet specific crosstalk between cells and the surrounding milieu.^25^ Therefore, there are specific considerations for 3D approaches based on the processes of metastasis,^26^ making this review timely and useful.

## Paradigm shift: from 2D to 3D

Understanding the underlying cellular events during bone metastasis is a mandatory requirement for development of appropriate and efficacious metastatic cancer models. Following this paradigm, studies of bone metastasis traditionally utilised 2D cultures to tackle the maldistribution of nutrition that leads to proliferation inhibition of the studied cells. Cells in 2D cultures are introduced to excessive nutrients and oxygen through an increased exchange area, which compels these cells to immortalise instead of suffering programmed apoptosis after multiple in vitro passages. Further, cells that adhere to flat and rigid 2D substrates experience loss of cell polarisation, along with a modified amount, configuration and composition of ECM proteins. This is of vital significance; the ECM is a crucial component of metastatic cancer microenvironments because the ECM exerts a functional influence on cancer cell proliferation, migration and apoptosis by modulating a series of soluble cytokines and signalling pathways.^27^ Additionally, the ECM communicates with cellular components frequently in vivo, while the lack of ECM results in diminished crosstalk between metastatic cells and the surrounding microenvironment that can lead to the disruptive establishment of bone metastasis. Moreover, monolayer cell cultures misrepresent whole-solid tumours for therapeutic drug development because 2D-cultured cells are normally immortalised and particularly vulnerable to drugs targeting rapidly dividing cells. In contrast, the tumour mass in vivo comprises a wide range of cellular states, including rapidly growing, stagnated, necrotic and apoptotic cells, which is a significant divergence from 2D cancer bone metastasis models.

To overcome these experimental constraints, researchers have developed multiple viable 3D approaches to model bone metastasis while maintaining reproducibility and enhancing complexity. These models have the following merits, which 2D approaches lack: (a) non-toxic, good biocompatibility that will not trigger subsequent inflammation or an immune response; (b) appropriate biodegradation rates that suit tissue regeneration rates; (c) easy processing while retaining hardness and mechanical strength to a certain degree, with a stable structural appearance; (d) adequate porosity and specific surface area; and (e) desirable surface activity to maintain viable cancer cell morphologies and phenotypes.^28–31^ Unlike 2D cultures, 3D biosystems have a consistent spatial framework, which helps to directly correlate structures with functions. In addition, in 3D models, the components of the supporting matrix (such as a collagen scaffold and hybrid substrate) can simulate the loose or dense connective tissues surrounding host cells, thus creating a desirable milieu for studying the migration and invasion of metastatic cancer cells. The cellular phenotypes and signal transduction in 3D platforms are more similar to in vivo conditions than are those in 2D cultures, providing a reliable re-establishment of vivid crosstalk between metastatic cancer cells and neighbouring matrices.^32^ Moreover, 3D systems promote rich cell–cell and cell–ECM interactions, which helps in the precise recapitulation of cancer bone metastasis at the tissue, cellular and molecular levels. In addition, multiple studies have illustrated that 3D approaches lead to a comprehensive understanding of the chemosensitivity of cells to therapeutic agents since these models represent actual solid tumour structures that consist of an inner necrotic core and an outer shell of proliferative metastatic cancer cells, contributing to a concentration gradient that is lowered from outside to inside. This gradient often hampers treatment effects, and a representative gradient model would be instrumental in developing clinically effective drugs. However, despite the aforementioned advantages of 3D models for studying bone metastasis, the ability of each individual biosystem to simulate a specific host microenvironment is quite different. For instance, collagen scaffolds lack dense tissues, and cell-derived scaffolds have larger porosity and are thinner than tissue-derived scaffolds, which enable cancer cells to metastasise relatively quickly compared with the host in vivo microenvironment. Therefore, these conditions require researchers to devise corresponding customised experimental toolkits for distinctive research purposes and to develop novel strategies to meet the demands of evolving studies.

## Three-dimensional approaches to model cancer bone metastasis

**Fig. 2:**
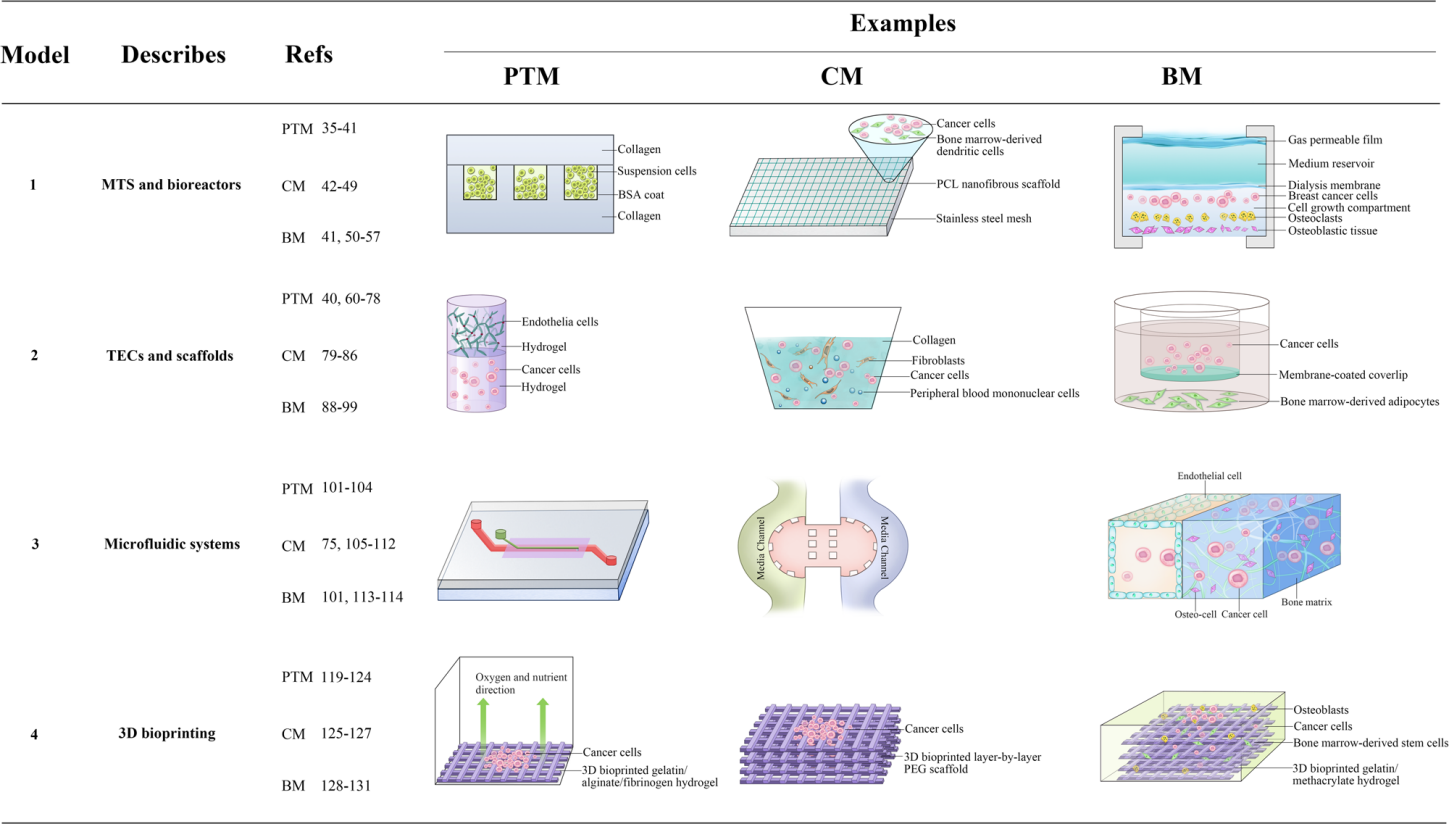
Examples of in vitro engineering 3D approaches in modelling the dynamic microenvironments of cancer bone metastasis referenced in this review

As mentioned above, it is important to re-establish the 3D cellular architecture and surrounding microenvironment for in vivo*-*like bone metastasis models. Based on the characterisation of the unique crosstalk between osteotropic cancer cells and the exterior microenvironment as well as diverse methods for 3D scaffold fabrication, we summarised several inspiring types of 3D approaches for recapitulating key cellular events within the three cancer microenvironments (PTM, CM and BM). Figure 2 shows these formats and offers selected references.

### Multicellular tumour spheroids (MTSs) and bioreactors

Mixed cell MTS systems have become a promising approach since their development in the 1970s. MTSs can form cell aggregates with necrosis inside and an uneven distribution of growth cytokines and metabolic needs, resembling the natural characteristics of metastatic solid tumours.^33,34^ Furthermore, the combination of MTSs with relevant bioreactors recapitulates specialised and controllable metastatic microenvironments that consider the sophisticated crosstalk within and between cells.

#### Modelling cellular transformation in the PTM

Within the PTM, cellular transformation induces infinite proliferation, altered morphology and enhanced invasiveness of malignant cells. Accordingly, various 3D MTSs and MTS-based approaches have been employed to model this process. Studies have shown that BC cell MTSs have increased malignant stemness and differentiation abilities with branching morphogenesis, loss of polarity, luminal filling and active cell invasion,^35^ which allow for assessment of therapeutic drugs, radiation and miRNA.^36–38^ In addition, the MTS applications can be combined with numerous bioreactors, including liquid overlay, spinning flask and gyratory rotation, hanging drop and suspension culture systems.^39,40^ The rotating wall vessel (RWV) has emerged in the microgravity milieu for constructing 3D cell tumour aggregates in which cells experience near-laminar flow conditions to survive in a suspension state in medium.^41^

#### Modelling immune-survival in the CM

MTS cultures of BC cells and alloimmune spleen cells was first used to demonstrate that immune cells are capable of penetrating tumour aggregates to kill interior malignant cells.^42^ However, devoid of stimulation by cytokines and monoclonal antibodies, cancer cells cultured in MTSs showed immune-resistance against NK cell-mediated death.^43,44^ Additionally, antigen presentation plays a vital role in immune attack, leading to the threshold of immune responses against cancer.^45^ This effect, which is mediated by antigen-presenting cells (APCs), such as dendritic cells, monocytes and macrophages, was re-established via co-culture of APCs in MTSs, which demonstrated a transition in morphology, invasiveness and differentiation that resulted in a decreased capacity for antigen presentation and promoted cancer malignancy.^46–49^

#### Modelling the BM

**Fig. 3:**
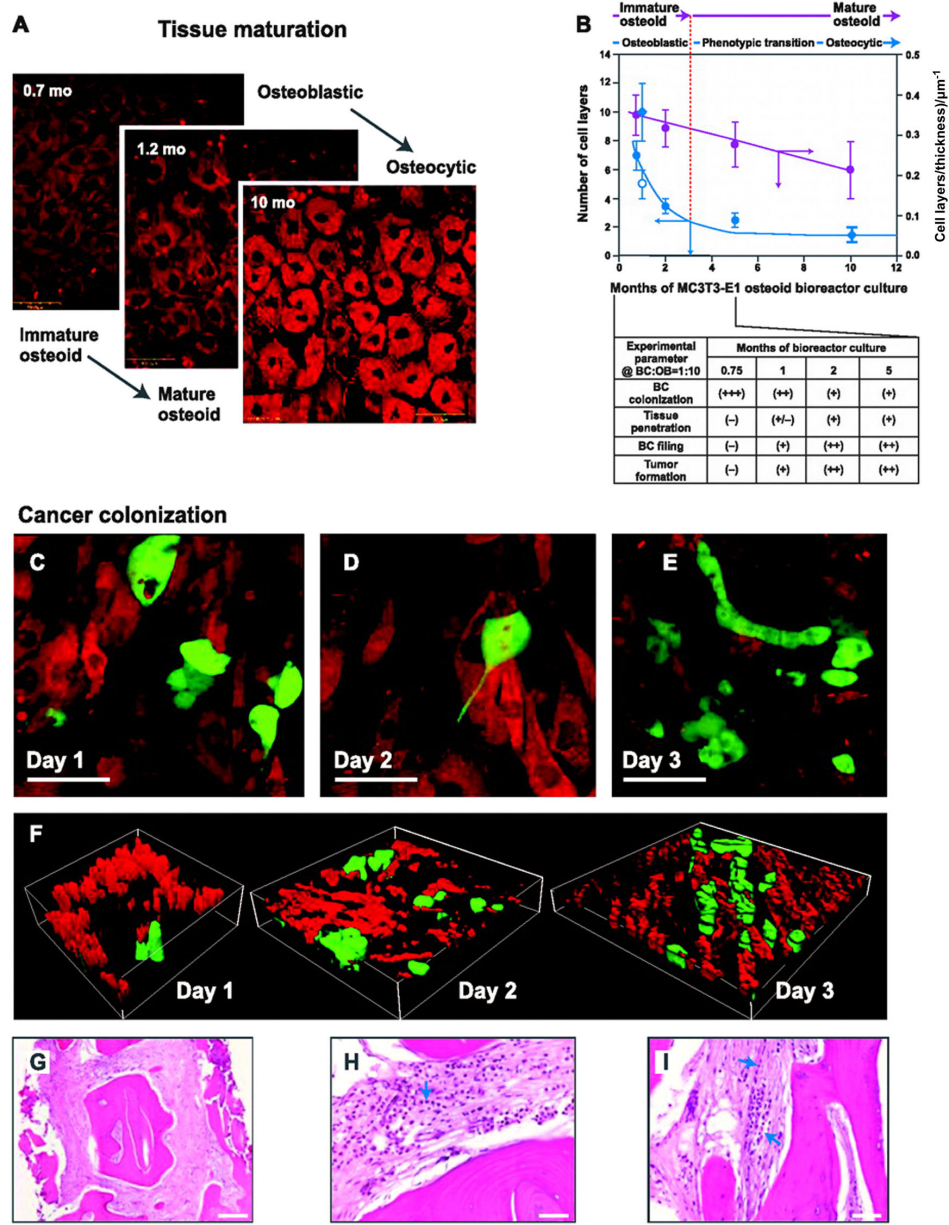
**a** Osteogenic tissue maturation in the bioreactor recapitulates development of native bone by systematic and reproducible phenotypic maturation of preosteoblasts through mineralising osteoblasts to terminally differentiated osteocytes. **b** MC3T3-E1 cells produce and mineralise a thick, engulfing ECM that slowly decreases in thickness and number of cell layers through progressive apoptosis to a final stable state exhibiting no sign of tissue necrosis over 10 months of continuous culture (graph). **c**–**f** Interaction of MDA-MB-231^GFP^ human cancer cells (green, GFP) with osteogenic tissue (red, osteoblasts; black, ECM) depends on tissue maturity (**b**, table) and exhibits stages of cancer cell adhesion (**c**), penetration (**d**) and alignment of cancer cell into files (**e**) that are reminiscent of events observed in pathologic tissue. **f** Filing is especially evident in corresponding 3D confocal reconstructions. **g**–**i** For comparison, Indian Filing is shown in a section from bone (solid pink) with metastatic breast cancer (rows of cells with dark purple nuclei). The scale bars in **a**, **c** and **d** represent 50 μm, the scale bar in **f** represents 100 μm, the scale bar in **g** represents 200 μm, and the scale bars in **h** and **i** represent 50 μm. Reprinted with permission from ref. ^55^ Copyright (2009) American Association for Cancer Research (AACR) Publishing Group

There are few reports on using MTS culture alone to model the BM, which is likely because this simplistic intercellular communication model fails to recreate the complex cancer-calcified matrix (bone) interplay. Thus, osteoblast-secreted matrices from MTSs have been used for modelling the BM. These matrices will be described in more detail in the “Tissue engineering construct” section. Here, MTSs of PC cells, osteosarcoma cells, osteoblast cells and stromal cells were generated for application in an RWV bioreactor, demonstrating that PC cells could induce permanent morphologic alterations in co-cultured osteoblasts and stromal cells, which stimulated the proliferation and invasiveness of PC cells.^50^ Compared with regular MTS culture, RWVs produce large batches of cell aggregates with a limited necrotic core^51^ and can be used to establish osteotropic tumours with areas of limited proliferation and decreased drug sensitivity.^41,52^ Moreover, unlike RWVs that minimise natural gravity, other bioreactors re-establish the natural forces within the BM. Using a specialised compartmentalised bioreactor, osteoblast MC3T3-E1 cells were co-cultured with BC MDA-MB-231 cells, and the results showed that less mature osteoblasts could bolster BC localisation more readily. Co-culture of BC cells further induced activation of osteoblasts and osteoclasts with reduced expression of osteocalcin and increased IL-6 levels, resulting in remarkable bone matrix degradation.^53,54^ This biosystem was implemented in another specialised bioreactor that allowed for maturation of 3D multi-cell-layer osteogenic tissue to study the metastatic interactions between cancer cells and bone^55^ (Fig. 3). BC cells tended to invade through the thick bone matrix after the early stages of metastasis, leading to matrix destruction. Mastro et al.^55^ showed that BC MDA-MB-231^GFP^ cells could invade thick osteoblast-embedded ECM to form “Indian files”, chains depicted as infiltrating lobular or metaplastic BCs. Osteoblasts were found to align in parallel with the cancer cells and exhibited increased inflammatory cytokine production. In addition, subsequent stages of invasive BC cells developed enhanced chemotaxis towards activated osteoclasts to form tumour colonies, accompanied by decreased osteoblastic tissue thickness and increased differentiation of osteoclast precursors.^56,57^ These bioreactors support MTS cultures (cancer cells, osteoblasts and osteoclasts) and thus contribute to the re-establishment of a “vicious cycle” for studying osteobiology and osteopathology in BM models of bone metastasis in vitro.

### Tissue engineering constructs (TECs) and scaffolds

TECs and scaffolds are the most widely used 3D approaches for modelling the microenvironments of bone metastasis because of their relatively available sources and convenient manipulation.^58,59^ Herein, scaffolds applied for TECs are based on natural materials or synthetic polymers, which have distinct advantages and disadvantages. Natural construct scaffolds are similar to natural ECM and possess inherent biocompatibility that is derived from the use of non-toxic substances. Synthetic constructs can be obtained from medical-grade purified molecules and manufactured under personalised guidelines. However, the drawbacks of natural and synthetic materials are obvious. Natural constructs have weak mechanical properties, uncontrollable degradability and between-batch inconsistency. Synthetic constructs carry the risk of loss of intrinsic bioactivity and modulus stiffness over the incubation period, leading to degradation of these compounds into toxic decomposition products. Therefore, it is vital to weigh both the advantages and disadvantages to establish the most suitable 3D TEC models for bone metastasis studies; this effort has led to the combination of natural molecules and synthetic scaffolds, which is a promising strategy for modelling bone metastasis.

#### Modelling cancer-related angiogenesis in the PTM

Natural materials, such as collagen, fibrin and laminin-rich basement membrane extract (BME/Matrigel), which originate from the ECM, have advantages in providing natural sites for cell attachment and signalling conduction to promote endothelial cell proliferation, migration and formation into capillary-like microstructures.^60–63^ This type of ECM-derived biomaterial has long been the most widely used approach in cancer-related angiogenesis studies. For instance, osteotropic BC MCF-7 cells cultured in 3D Matrigel have increased expression of VEGF, bFGF and IL-8, which are the main mediators of endothelial cell recruitment and differentiation and thus promote angiogenesis.^64^ Culturing endothelial cells on collagen matrices with cancer cells and fibroblasts leads to the formation of branching microvasculature, indicating that cancer cells and fibroblasts embedded in collagen can trigger migration and differentiation of endothelial cells with elevated expression of VEGF.^60,65^

Recently, the utilisation of semi-synthetic, naturally derived, protein-based hydrogels, such as alginate- and collagen-based hydrogels, has surpassed the use of wholly synthetic scaffolds, such as poly(lactide-co-glycolide) (PLG) and poly(lactide-co-glycolide acid) (PLGA), in modelling angiogenesis since synthetic scaffolds alone lack bioactive growth factors. Thus, co-culture of osteotropic PC cells and endothelial cells on hydrogel decorated with IKVAV and GFOGER peptides was found to lead to increased vascular cell infiltration with significant blood tube outgrowth.^66,67^ Poly(ethylene)glycol (PEG)-based hydrogel-supported LC cells induce the release of proangiogenic TGF-β to stimulate the neovasculature of endothelial cells, resulting in further significant tumour progression compared to hydrogels without vascular cells.^68^ Moreover, co-culture with endothelial cells or embryoid bodies contributed to the directional migration of endothelial cells towards the tumour, leading to the establishment of a tubular structure and luminal network.^69,70^ Another significant 3D biosystem is the combination of a human arterial ring (hAR) with Matrigel in which PC LNCaP cells facilitate angiogenesis by stimulating the release of proangiogenic factors from the hAR to sustain angiogenesis in the absence of an exogenous stimulus.^71^

#### Modelling cellular transformation in the PTM

Using natural matrices that mimic the structural and mechanical properties of the ECM, Oyanagi et al.^72^ found that LC A549 cells treated with TGF-β in a 3D collagen matrix had EMT traits, including extended microtubule-based protrusions and increased migration ability.^72^ Additionally, BC cells cultured in a collagen matrix developed irregular cellular polarity, increased cisplatinum resistance and enhanced metastasis ability, which can be attributed to the altered expression of integrins, proangiogenic factors and EMT proteins in 3D cultures.^40,73^ Fibroblast-derived matrices produced by cancer-associated fibroblasts have also been used to culture osteotropic cancer cells, inducing a significant change in cell morphology, proliferation and invasiveness compared to 2D cultures.^74^ In addition, artificial synthetic scaffolds, such as hyaluronic acid (HA) hydrogels, can support a finger-like architecture of PC cells, allowing for quantification of cell size, shape and convergence, which support the metastatic ability of PC cells.^75^ Additionally, PLGA and poly(ε-caprolactone) (PCL) scaffolds can support the 3D culture of malignant cells, enabling investigation of microenvironment stimuli acting on these cells, including inhibition of proliferation and invasion with cytotoxic drugs.^76,77^ PC cells cultured in a chitosan-HA matrix developed stem-like features and EMT attributes compared to those in a 2D system, revealing that HA likely promotes the metastasis, EMT and drug resistance of PC cells, followed by the activation of downstream signalling involved in cancer malignancy.^78^

#### Modelling immune-survival in the CM

Considering the actual purposes of CM modelling, various natural or synthetic scaffolds were combined with MTSs. A 3D spheroid on collagen gel gave cancer cells the ability to promote tumour-associated macrophage (TAM) transition,^79^ providing an immune-regulatory potential for evaluating the in vivo*-*like immunotherapeutic effects of immune cells compared with 2D cultures.^80,81^ When cultured on a 3D chitosan-alginate scaffold, BC cells expressed IFN-γ and CCL21 cytokines to induce the recruitment and infiltration of T cells into a MTS, providing a promising CM model for assessing T-cell function.^82^

#### Modelling directional migration in the CM

Collagen-based scaffolds supported directional metastasis of BC cells with invasive phenotypes compared to Matrigel or RADA16 peptide scaffolds, which could be enhanced by the increased collagen density.^83–85^ A bovine serum albumin (BSA) scaffold with fibronectin (FN) showed desirable BC cell adhesion and migration compared with the BSA scaffold alone.^86^

#### Modelling the BM

Notably, since osteoblast differentiation and mineralisation leads to the formation of mature bone,^87^ it is feasible to use osteoblasts to model 3D osteoblastic matrices for cancer cells in the BM. Mineralised ECM released from human osteoblasts supports cellular progression of metastatic BC cells by establishing potent attachment forces between BC cells and osteoblasts, which are mediated by the production of β_1_-integrin, osteopontin and matrix-metalloproteinase (MMP) 2 and 9 in metastatic BC cells.^88^ Similarly, another decellularized osteoblastic mineralized matrix indicated that a collagen-I-rich fibril matrix with various ECM proteins could provide a compatible microenvironment for PC cells to induce adhesion, proliferation and metastasis.^89^ Nevertheless, such models of osteoblast-secreted matrices are considered difficult to manufacture on a large scale and have insufficient stiffness, resulting in formidable challenges for translational applications. A 3D approach for engineering osteoblastic bone tissue was developed, combining the advantages of osteoblastic matrices with a medical-grade polymer system. Polycaprolactone-tricalcium phosphate (PCL-TCP) scaffolds wrapped with induced mineralized osteoblast sheets were devised for co-culture with PC cells, revealing the compatible colonization of PC cells onto engineered bone with metastasis-associated molecules, such as MMPs, prostate specific antigen (PSA) and steroidogenic enzymes.^90^ When PC cells were pre-embedded in hydrogel to synthesise the PCL-TCP scaffold, engineered bone decreased the ingrowth of PC cells. Importantly, PC cells produced osteomimetic cell phenotypes, as exemplified by upregulation of bone and vasculature markers and activation of TGF-β downstream genes.^91^

Although numerous 3D osteoblastic approaches have been used to model osseous or osseous-like scaffolds of the BM, natural and synthetic TECs can also recreate the osteomimetic response of osteotropic cells with other cell types that constitute the bone microenvironment. For example, various 3D collagen gels (collagen-based glycosaminoglycan or nanohydroxyapatite) have been used to simulate biological intercellular events between metastatic cells and bone marrow-derived cell types, such as adipocytes, mesenchymal cells and endothelial cells, to study the directional bone-homing and proliferative effects of malignant cells.^92–94^ Fitzgerald et al.^94^ demonstrated that three types of collagen-based scaffolds (ColGAG, S200 and S500) remained structurally stable after 14 days of PC cell co-culture, indicating the potential application of collagen-based ColGAG, S200 and S500 scaffolds for PC implantation up to 14 days. Moreover, transplantation of bone marrow stromal cells (BMSCs) in silk scaffolds and BMP-2 stimulation of mouse mammary pads demonstrated the bone-targeting effects of metastatic BC cells in vivo*.*^95,96^ BC 4T1 cells cultured in 3D a collagen-glycosaminoglycan scaffold developed mineralization sites with increased levels of collagen-I and bone sialoprotein when stimulated with exogenous phosphate and BMP-2.^97^ Stromal and endothelial cells were used on the fibrin scaffold to develop a 3D BM for MM bone metastasis, recapitulating the 3D metastatic bone niche for MM cells.^98^ Mesenchymal progenitor cells were transplanted onto a tubular composite scaffold supplemented with BMP-7 for PC bone metastasis, demonstrating the morphogenesis of physiological bone tissues and the directional migration of PC cells to humanised bone tissues with cancer-induced bone lesions.^99^

### Microfluidic systems

In vivo, osteotropic cancer cells experience a continuous fluidic microenvironment in the PTM, CM and BM. Hence, it is important to consider the fluid stresses that cancer cells withstand when modelling metastatic microenvironments. Microfluidic systems that provide high-throughput and high-content approaches^100^ have received significant attention in the 3D recapitulation of cancer bone metastasis. Such systems allow for effective high-resolution and high-sensitivity analysis with a small number of cells and reagents to create in vivo*-*like 3D flowing conditions in vitro.

#### Modelling cancer-angiogenesis in the PTM

Three-dimensional bioengineered microfluidic scaffolds have well-proportioned fluid stress, leading to balanced dissemination of cancer cells with supportive cells within uniform-sized cell spheroids^101^ that can maintain malignant angiogenesis in the PTM. One example is the co-culture of BC cells with endothelial cells in a microfluidic system in which low fluidic conditions contributed to the convergence of endothelium with a functional microchannel lumen and enhanced cancer malignancy.^102^

#### Modelling cellular transformation in the PTM

Cancer cells cultured in a microfluidic biosystem developed spheroid cell aggregates exhibiting decreased levels of epithelial markers, such as CD326, and elevated expression of mesenchymal markers, such as N-cadherin, vimentin and fibronectin, compared with cells cultured in the 2D bio-platform.^103^ Additionally, a gel-free nanoscaffold provided LC cells with a 3D milieu for TGF-β-induced EMT transformation morphologically and genetically,^104^ indicating potential re-establishment of the EMT process using a micropattern approach.

#### Modelling directional migration in the CM

The 3D approaches for the CM also require recapitulation of the kinesiology of osteotropic cancer cells, which can generally be classified as transendothelial movements (intravasation and extravasation) and directional migration within the circulatory system. Penetration through endothelium depends on the active interaction between cancer cells and stromal cells, and the directional migration and invasion of malignant cells rely on the chemoattraction of homing sites.^105,106^

To date, researchers have primarily taken advantage of microfluidic systems to establish dosage gradients of chemokines and to simulate the shear stress and interstitial flow that the endothelial network imposes during transendothelial migration to model the dynamic bioactivity of cancer cells in the CM.^75^ The hydrodynamics of a microfluidic chip enable assessment of tumour cell alterations at morphological, genetic and protein levels related to membrane rearrangement and subsequent migratory activity.^107,108^ Cancer cells can transmigrate through the endothelial monolayer into the collagen gel within the microfluidic platform, showing tightly regulated and well-modelled extravasation of cancer cells, as evidenced by the ability to target the regulation of CXCL12, epidermal growth factor (EGF), interstitial flow, cell morphology and matrix stiffness.^109–111^ Additionally, a cell-based microfluidic chip was devised in which both the intravasation and extravasation processes in metastasis could simultaneously reappear, demonstrating the detachment of malignant cells from the Matrigel matrix under sheer stress in the intravasation chamber, followed by the attachment to adhesion molecules expressed by endothelial cells in the extravasation chamber.^112^

#### Modelling the BM

**Fig. 4:**
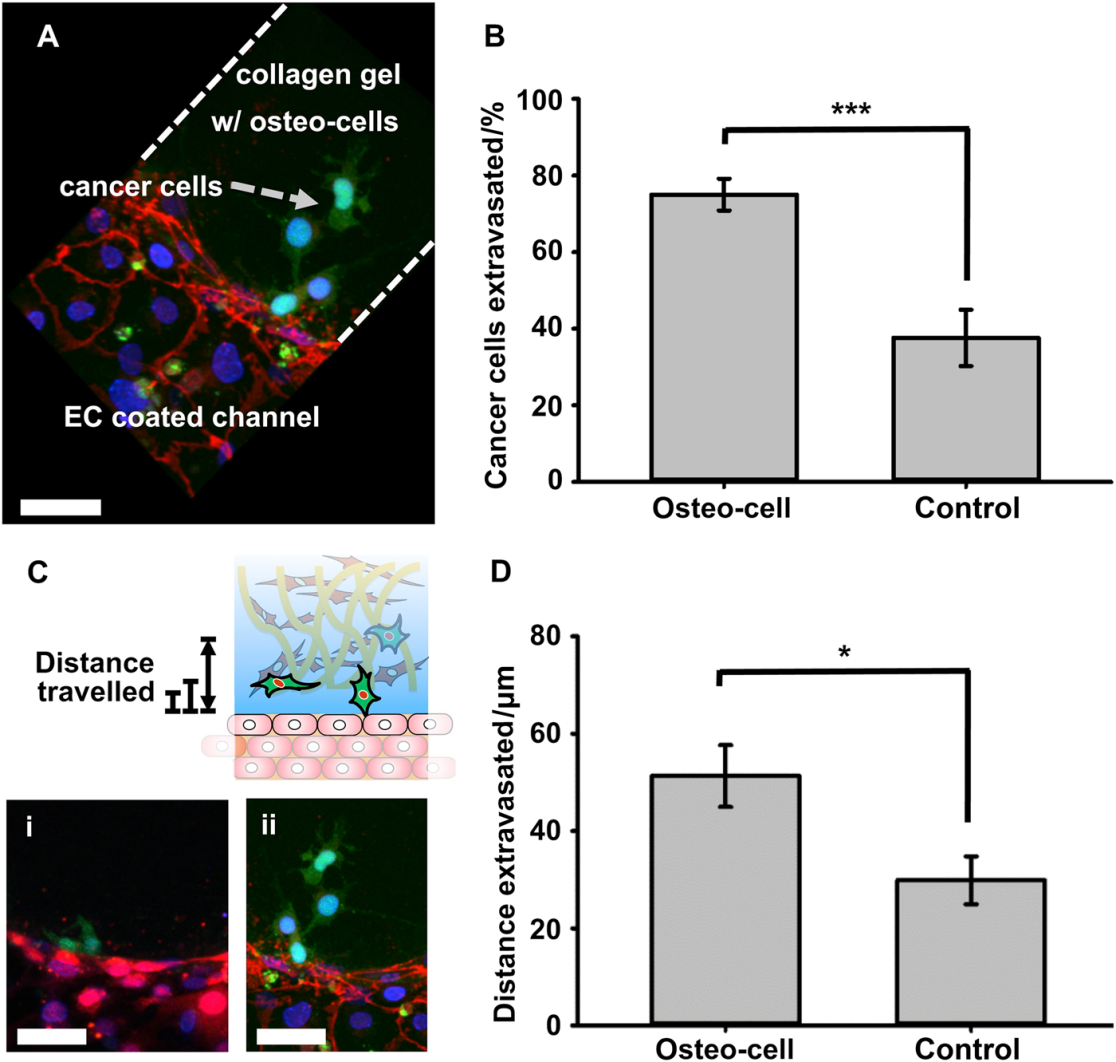
Extravasation of cancer cells into the collagen gel matrix with and without osteo-differentiated hBM-MSCs (labelled as osteo-cells). **a** Three-dimensional confocal reconstruction shows MDA-MB-231 cancer cells (GFP) transmigrated across the endothelial monolayer into the collagen gel containing osteo-differentiated hBM-MSCs. VE-cadherin (red) and DAPI (nuclei, blue) staining. **b** Average percentage of extravasated cancer cells was significantly higher (***P < 0.005) in the collagen gel with osteo-differentiated hBM-MSCs. **c** Projected images show extravasated cancer cells (GFP) travelled farther into the osteo-cell conditioned microenvironment (ii) compared to the collagen gel-only matrix (i). Cells were stained with (i) DAPI (nuclei, blue) and (ii) VE-cadherin (red) + DAPI (nuclei, blue). HUVECs were RFP labeled. **d** Average distance travelled by extravasated cells into the gel matrix increased significantly in osteo-cell conditioned microenvironment (*P < 0.05). Scale bars represent 50 μm. Reprinted with permission from ref.^114^ Copyright (2014) Elsevier Publishing Group

A microfluidic device was applied to modelling the BM because this device can mimic the bone milieu for investigating dynamic cancer metastasis. To construct an organ-specific 3D microfluidic approach for osteotropic cell metastasis, primary and mineralized bone mesenchymal stem cells (BMSCs) and endothelial cells were tri-cultured, leading to the establishment of a vascularises bone-like microenvironment for studying the extravasation rate and microvascular permeability of BC cells.^113^ Similarly, a vascularises microfluidic system of BMSCs and endothelial cells was devised, followed by the subsequent introduction of BC MDA-MB-231 cells into vessel-like channels, resulting in a viable formation of micrometastases of extravasated cancer cells in the attractive matrix^114^ (Fig. 4). Moreover, by combining the microfluidic bio-platform with tri-cultured cell spheroids comprising osteoblasts, endothelial cells and PC cells, this flowing chip enabled real-time monitoring of each spheroid and the interior PC cells, indicating the curbed proliferation of PC cells without jeopardising the cell viability. This system could be employed to more accurately model the in vivo growth patterns of malignant cells within the metastatic BM.^101^

### Three-dimensional bioprinting

Recent progress in 3D bioprinting has triggered substantial innovations in the construction of sophisticated 3D functionalized living tissues and organs, including bones,^115–117^ and there is increasing interest in re-establishment of the dynamic microenvironments of bone metastasis. The use of bio-inks, which consist of cell mixtures in the form of compatible gel-like material, is expected to be used for depositing and generating 3D scaffolds that mimic spatial metastatic microenvironments at high resolution.^118^ However, compared with other 3D approaches commonly used for engineering dynamic microenvironments, there are limited reports on the use of 3D bioprinting to evaluate cancer bone metastasis, and bioprinting might be an attractive realm for future research and translation.

#### Modelling cancer-angiogenesis in the PTM

Because 3D bioprinting fibres embedded in hydrogels can generate microvessels, patterning cells and biomaterials for cancer-angiogenesis in PTM has received much attention.^119^ In one study, a sacrificial template was deposited with a bioprinter in a random pattern similar to the host vasculature network, followed by placement of the cast hydrogel around the template. After removal of the template, there were porous microchannels within the hydrogel. Along with these microchannels, endothelial cells could be internalised to faithfully form a functionalized vasculature system. Based on these results, a microchannel system with human umbilical vein endothelial cells (HUVECs) and seeded with MTSs showed that MTSs were remodelled and exhibited potential angiogenesis.^120^

#### Modelling cellular transformation in the PTM

By co-culturing LC cells with agarose and alginate, which served as scaffold materials to mimic the growth milieu, 3D bioprinting allowed for desirable proliferation of LC cells, which were able to migrate and invade into the adjacent scaffold.^121^ Furthermore, the in vivo orientation between stromal cells and cancer cells could be recapitulated via 3D bioprinting in vitro by depositing cell suspensions onto the basement in a predefined pattern, revealing a feasible co-culture platform that was amenable to controlling cell droplet size, density and malignant morphology. The cells in this system had a more metastatic phenotype than did those in 2D cultures.^122,123^ Additionally, BC cells cultured in a custom-built bioprinting platform demonstrated more in vivo*-*lik*e* and uniform cellular spheroids in situ on a hydrogel substrate, representing a controllable and high-throughput approach for modelling PTM.^124^

#### Modelling immune-survival in the CM

It was demonstrated that a co-extrusion bioprinting model could be used to study the interaction between BC cells and macrophages, contributing to the development of paracrine cycle-stimulated migration and extravasation of BC cells.^125^ The results further showed that macrophages could escape from microvessels to communicate with BC cells, providing a convenient model for studying immune-survival of cancer cells in the CM.

#### Modelling directional migration in the CM

Three-dimensional bioprinting is advantageous for providing a CM matrix that supports the migration of osteotropic cells during bone metastasis. A honeycomb structure in a hydrogel was created with 3D bioprinting to simulate the vasculature in vitro for evaluating the migration of cancer cells, which showed that a reduced width of bioprinted capillaries increased the migratory velocity of cancer cells.^126^ In addition to the vessel diameter, a bioprinted PEG scaffold showed that substrate stiffness and cell morphology significantly impacted the migration speed of malignant cells.^127^

#### Modelling the BM

**Fig. 5:**
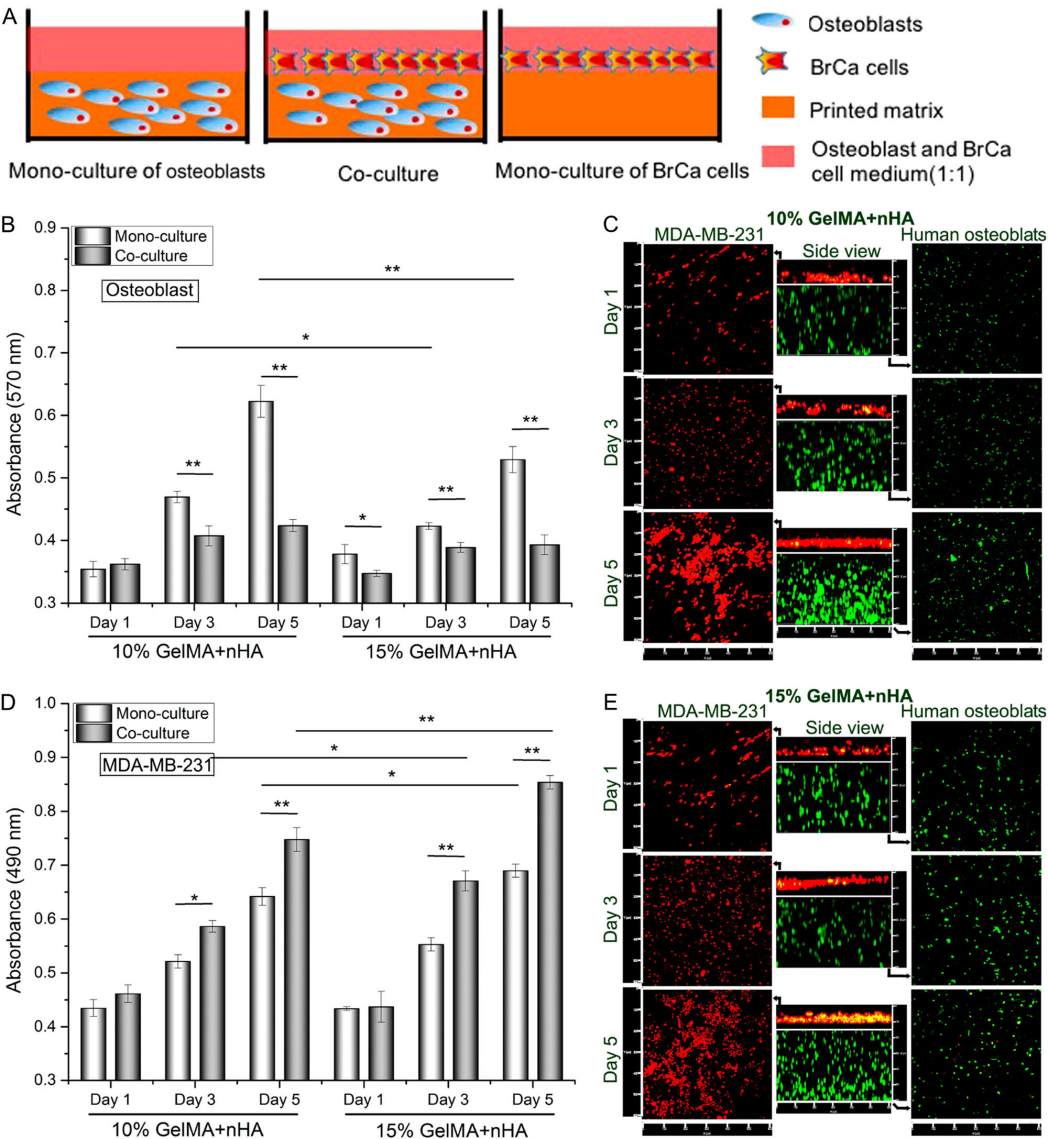
**a** Schematic diagram of osteoblasts and BrCa cells mono- and co-cultured in the 3D bioprinted matrix. Proliferation of **b** osteoblasts and **d** BrCa cells mono- and co-cultured in the 3D bioprinted matrix after 1, 3 and 5 days. *P < 0.05, **P < 0.01. **c**, **e** Confocal micrographs of osteoblasts and BrCa cells co-cultured in the 3D bioprinted matrix after 1, 3 and 5 days. The middle columns in **c**, **e** represent the cross-sectional views. Osteoblasts and BrCa cells were stained by Cell Tracker Green CMFDA dye (green) and Orange CMTMR dye (red), respectively. Reprinted with permission from ref.^129^ Copyright (2016) American Chemical Socie1ty

Comparison of typical 3D natural and synthetic scaffolds in terms of the substrate modulus and pore size indicates that 3D bioprinting produces more in vivo*-*like trabecular bone for modelling the BM in bone metastasis. A template-fused deposition modelling strategy was used to generate a tuneable porous matrix with skeletal parameters similar to in vivo cancellous bone.^128^ Furthermore, when co-cultured with BMSCs, this bioprinting system enabled recapitulation of enhanced osteogenesis with increased matrix stiffness and decreased pore diameter, which successfully modelled the BM during bone metastasis. Additionally, BMSCs or osteoblasts encapsulated in gelatine methacrylate (GelMA) and polyethylene glycol hydrogel with nanocrystalline HA were used to constitute the bone matrix for bioprinting. The subsequent co-culturing of BC cells potentiated cancer cell ingrowth and enhanced the expression of VEGF and IL-8 due to stimulation of BMSCs or osteoblasts.^129,130^ Zhou et al.^131^ showed that the proliferation of BC cells was enhanced due to osteoblast co-culture, while the growth of osteoblasts was inhibited because of BC co-culture, especially in 3D bioprinting scaffolds (10%/15% GelMA + nHA) (Fig. 5). Moreover, BC cells formed spheroids with potent metastatic characteristics.^131^ However, this effect was alternatively followed by an inhibition of osteoblastic mineralization in 3D bioprinted cultures.

## Conclusion and future perspectives

Three-dimensional engineering approaches provide controllable and representative toolkits to model the dynamic microenvironments of bone metastasis with the incorporation of functionalized cells, ECM, growth cytokines and other biochemical stimuli. These models allow for a reliable recapitulation of cancer metastatic microenvironments and facilitate the evaluation of cell–cell and cell–ECM crosstalk and thus can aid in elucidating malignant metastatic mechanisms and will enable the development of novel therapeutic strategies.^132^

However, major challenges and limitations remain. First, none of the current models recapitulates the entire process of metastasis in a single culture system. Notably, osteotropic cancer cells that metastasise from the primary site to distant bone undergo a sequential and closely related multistep process, and the cellular events cannot be separated. Second, advances in 3D cultures mainly employ immortalized malignant cell lines while neglecting to introduce patient-derived cancer cells. The use of personalised cells rather than cell lines could aid in the development of customized therapies and would guide the treatment of patients with bone metastasis more effectively.^19,133,134^ Third, unlike the spheroids of MTSs in vitro, in vivo tumours tend to establish various 3D appearances, such as cauliflower-like, ulcerative and irregular-shaped forms. This discrepancy is of vital significance because the stereo-architecture of tumours remarkably affects osteotropic cancers in terms of oxygen diffusion, cell adhesion, and drug sensitivity. Fourth, because each 3D approach has its own merits and demerits, a potential future trend is to combine diverse platforms to mimic extensive metastatic microenvironments. For instance, a microfluidic system was enhanced with 3D bioprinting to fabricate specialised, customized and flowing devices suitable for particular applications.^135^ Lastly, although osteocytes play significant roles during bone metastasis, reports concerning the combination of osteocytes with dynamic 3D approaches to model the BM of bone metastasis are rare. Indeed, this interesting practice requires further study. Overall, despite the inspiring advances in engineering 3D approaches to model the dynamic microenvironments of bone metastasis, there is significant room to improve 3D approaches for a better understanding of the underlying mechanisms involved in bone metastasis.
